# Unlocking the Potential: immune functions of oligodendrocyte precursor cells

**DOI:** 10.3389/fimmu.2024.1425706

**Published:** 2024-07-09

**Authors:** Amr Haroon, Harsha Seerapu, Li-Pao Fang, Jakob Heinrich Weß, Xianshu Bai

**Affiliations:** ^1^ Molecular Physiology, Center for Integrative Physiology and Molecular Medicine (CIPMM), University of Saarland, Homburg, Germany; ^2^ Center for Gender-specific Biology and Medicine (CGBM), University of Saarland, Homburg, Germany

**Keywords:** oligodendrocyte precursor cells, immune functions, immunomodulation, CNS injury, phagocytosis, multiple sclerosis, brain injury

## Abstract

Oligodendrocyte precursor cells (OPCs) have long been regarded as progenitors of oligodendrocytes, yet recent advances have illuminated their multifaceted nature including their emerging immune functions. This review seeks to shed light on the immune functions exhibited by OPCs, spanning from phagocytosis to immune modulation and direct engagement with immune cells across various pathological scenarios. Comprehensive understanding of the immune functions of OPCs alongside their other roles will pave the way for targeted therapies in neurological disorders.

## Introduction

**Figure 1:**
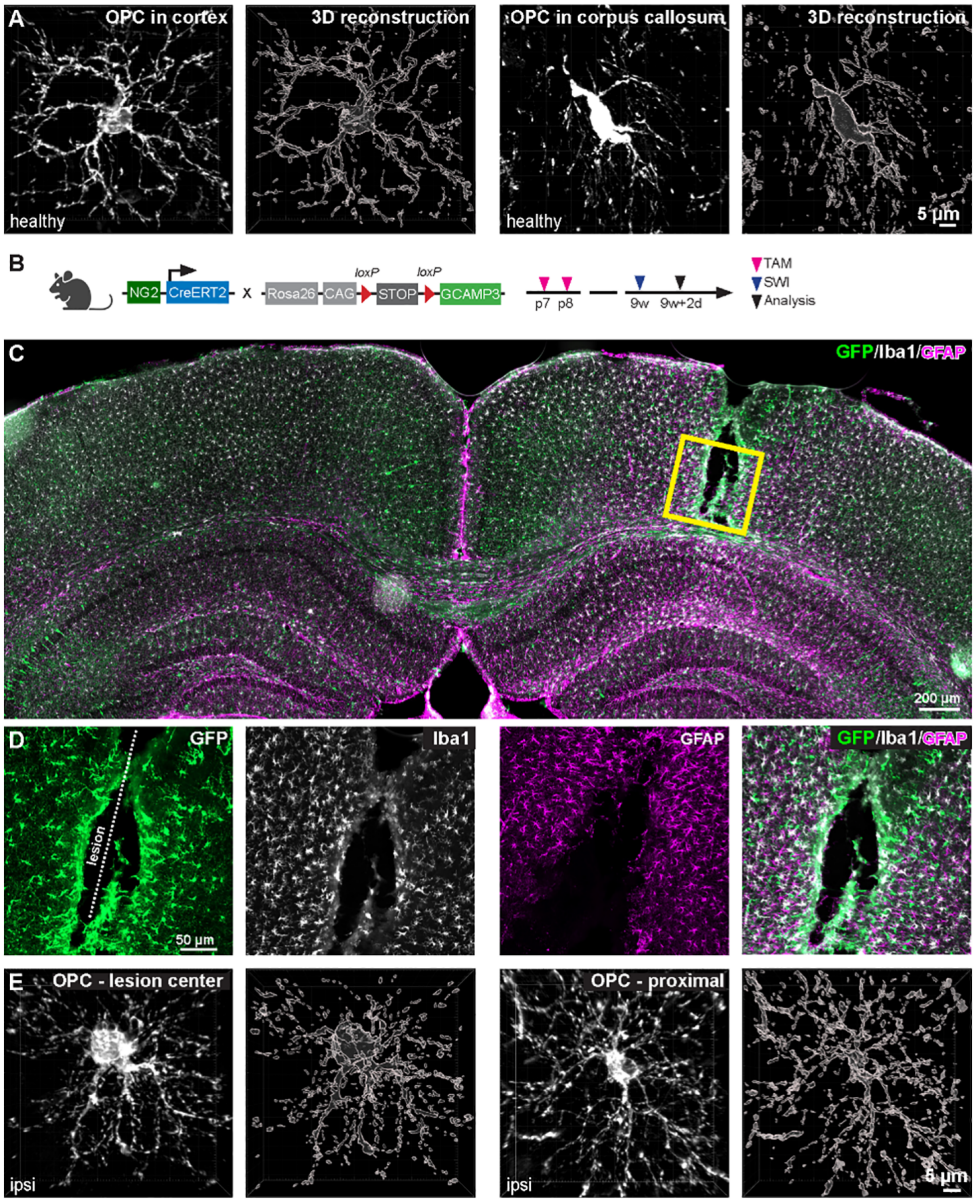
Morphological diversity of OPCs in healthy and pathological contexts. **(A)** OPCs in the corpus callosum (right pannel) are more elongated than in the cortex (left pannel). **(B)** Cortical stab wound injury (SWI) was induced in 9-week-old NG2-CreERT2 x GCaMP3 mice. Tamoxifen was administrated at postnatal day 7 and 8 (p7/8) to induce expression of GCaMP3 in OPCs. **(C)** Coronal brain slice was immunostained with GFP (for amplifying GCaMP3 signals in OPCs and OLs), Iba1 (microglia) and GFAP (astroglia) at 2 days post injury (dpi) of SWI. **(D)** Magnified images of boxed area in **(C)**. **(E)** At 2 dpi, OPCs increase morphological complexity at the center (left) and surrounding area (right) of the lesion. Scale bar in **(A, E)** = 5 µm, **(C)** = 200 µm, **(D)** = 50 µm. (unpublished data from Fang et al., 2023 (6)).

OPCs are the progenitors responsible for generating mature oligodendrocytes and facilitating subsequent myelination throughout life (1). OPCs are also considered NG2 glia due to their expression of NG2 (neural/glial antigen-2 or chondroitin sulfate proteoglycan 4) (2, 3). Platelet-derived growth factor receptor alpha (PDGFRα) is also widely used to identify OPCs, distinguish them particularly from NG2-expressing pericytes (4). Developmentally, OPCs in the mouse brain are generated in three waves: from the ventral forebrain, particularly from the medial ganglionic eminence (MGE) and the embryonic preoptic area (ePOA) around embryonic day (E) 12.5; from the dorsal brain, specifically the lateral and medial ganglionic eminences (LGE, MGE) around E15.5; and the subventricular zone during perinatal days (5). In adult, OPCs constitute approximately 5% of the total CNS population) (1), including both gray and white matter. Typically, OPCs display a stellate morphology characterized by small cell bodies and highly branched processes (**Figure 1A**). These processes frequently come into contact with various parts of neurons, such as the nodes of Ranvier (7, 8), presynaptic terminals (9, 10), and cell bodies (11), suggesting multiple ways of communication between OPCs and neurons (10, 12–15). In addition to direct neuron-OPC communication, OPCs indirectly shape brain circuits via many other pathways (15), including participation in BBB barrier function (16) and immune modulation (17).

In the CNS, immune function is mainly performed by microglia. As the resident immune cells, they actively and continuously survey the CNS microenvironment, using their processes to promptly detect signs of damage or foreign substances and eliminate them through phagocytosis (18). Serving as the primary responders to insults within the CNS, microglia release cytokines, chemokines, and other signaling molecules to orchestrate subsequent responses by other cell types. The activation of microglia can exhibit either pro- or anti-inflammatory characteristics depending on the context. Additionally, upon sensing threats or damage, microglia become activated and undergo changes in morphology. Mild activation leads to an increase in the complexity of their morphology, while intense activation triggers their transformation into an ameboid-like shape with enhanced phagocytic activity. Microglia also increase the expression of MHC I and II to facilitate the recruitment and activation of T cells in response to specific threats.

Notably, OPC processes are motile (19) and phagocytic (20, 21). Under pathological conditions, OPCs become hypertrophic (22, 23) (**Figures 1B–E**) and release and/or respond to cytokines (17, 24, 25). In multiple sclerosis (MS), OPCs also enhance the expression of MHC I and II molecules and interact with immune cells (24, 26). All of these studies provide strong evidence of the immune function of OPC. In this review, we delve into the immune functions exhibited by OPCs, expanding beyond their canonical role in myelination. We start with the phagocytic activity of OPCs. Then, we elaborate on the contribution of OPCs in wound healing and de-/remyelination with a particular focus on OPC-immune cell interaction. The comprehension of OPCs’ immunological roles carries substantial implications for neuroinflammatory diseases, neurodegenerative disorders, and the development of potential therapeutic interventions targeting the immune system within the CNS.

## Threat elimination through phagocytosis

Immune cells play a crucial role in engulfing foreign or harmful substances to safeguard the body—a function previously thought to be exclusive to certain cells until recently observed in OPCs (27, 28). Studies on primary OPCs isolated from rat brains revealed their phagocytic activity against various debris types, including myelin-rich debris from white and grey matter, as well as cell membrane lysate from cultured astrocytes, although their activity against amyloid beta was comparatively low (28). The mechanism underlying OPCs’ selective phagocytosis remains elusive, but one possibility is the involvement of low-density lipoprotein receptor-related protein 1 (LRP1) (**Table 1**), a transmembrane receptor known to bind directly to myelin basic protein (MBP) (29). Cells expressing LRP1 may selectively bind to debris expressing MBP, facilitating myelin-debris-specific phagocytosis. Inhibition of ligand-binding activity of LRP1 by antagonists like glutathione-S-transferase receptor-associated protein (GST-RAP) resulted in the inhibition of myelin debris uptake (29). However, the reasons for the preferential phagocytosis of astrocyte cell membrane over amyloid beta remain unclear. Moreover, it’s uncertain whether the substance preferentially phagocytosed by OPCs is dependent on the disease context. Addressing these open questions will necessitate further studies.

**Table 1:** Summaries of factors contributing to OPC-immune functions.

Factors	Cellular origin	Effect	Reference
LRP1	OPC	Phagocytosis of myelin derbris and presynaptic engulfment	(21, 29)
TGFβ2	OPC	Mediate microglia homeostasis and immune response via TGFBR2-CX3CR1 pathway	(17)
IL-1β, CCL2	OPC	Induction, activation and recruitment of CD4^+^ T cells and T helper cell type 1 (Th1)	(25, 30, 31)
Olig2	OPC	Overexpression of Olig2 enhance OPC migration and further differentiation in LPC-induced demyelination model	(6)
IFN-γ	- /T cells	Enhance OPC phagocytosis; Induce OPC quiescence; Increase MHC I and II expression in OPCs and further recruit T cells;	(24, 27, 32–34),
IL-4, IL-10	-	Reduce OPC phagocytosis; Inhibit OPC differentiation; Reduce MHC expression	(34)
TNF-α	-	Enhance OPC phagocytosis	(34)

The phenomenon of OPC phagocytosis extends beyond *in vitro* scenarios involving myelin debris or pathological contexts. Recent studies have unveiled that OPCs engulf axons and presynaptic terminals *in vivo* during brain development (20, 21). Axonal fragments, together with the presence of phagosomes, were observed within OPCs in the developing brain (20). Single-nucleus RNA-sequencing data showed that OPCs in the developing cortex express crucial phagocytic genes and neuronal transcripts, confirming their role in axon engulfment during development and suggesting their involvement in refining neuronal circuits throughout cortical maturation. Additionally, independent research has demonstrated OPC-mediated phagocytosis of presynaptic compartments in the developing visual cortex (21). This phagocytosis appears to be mediated by LRP1 (**Table 1**), as it was observed in some of OPC processes contacting synapses. Notably, OPC-mediated synapse phagocytosis plays a crucial role in neural circuit activity. In experiments where mice were subjected to darkness during the critical period (postnatal day 20–29) and subsequently exposed to light for only 10 hours, which induces neural activity, the number of synapses engulfed by OPCs increased compared to mice kept in darkness. Interestingly, the absence of microglia diminished OPC engulfing activity, suggesting a signal input from microglia for OPC phagocytosis (21). As OPCs form postsynaptic structures with neuronal axons, and neurotransmission via this communication triggers OPC differentiation into mature oligodendrocytes—cells that do not inherit synaptic connections (35, 36)—it is plausible that after receiving signals for differentiation, OPCs phagocytose presynaptic terminals projected to them, ultimately differentiate into mature oligodendrocytes. However, further studies are needed to validate this hypothesis.

An intriguing observation is that following phagocytosis, OPCs enter a quiescent state. Transcriptomic analysis has revealed that post-phagocytosis, OPCs downregulate genes associated with myelination and migration (28). The mechanisms underlying how OPCs decide their fate, whether to differentiate into oligodendrocytes or engage in phagocytosis, remain elusive. It is plausible that the pro-inflammatory factor IFN-γ, prevalent in various inflammatory microenvironments including demyelinated lesions, induces OPC quiescence (32). Blocking IFN-γ has been shown to rescue human OPC proliferation and differentiation (32, 33). IFN-γ triggers the upregulation of the transcriptional regulator paired related homeobox protein 1 (PRRX1) in human OPCs, leading to cell-cycle arrest *in vitro* (32). This suggests that precursor and immune functions may be mutually exclusive. However, a recent study demonstrated that triggered by anti-inflammatory cytokines (IL-4 and IL-10), primary OPCs reduced both differentiation and phagocytic activity (34) (**Table 1**). Conversely, pro-inflammatory cytokines like IFN-γ and TNF-α did not affect OPC differentiation but increased phagocytic activity (**Table 1**). This discrepancy might stem from the inability of these cytokines, although abundant in inflammatory conditions, to fully replicate the complex inflammatory microenvironment *in vivo*. There could be synergistic or antagonistic effects between various cytokines along with IFN-γ or IL-4. Therefore, a more comprehensive study is warranted to elucidate how OPCs determine their fate, whether to engage in phagocytosis or remain as active precursors.

## Glial scar formation and wound healing

After CNS injury, OPCs undergo rapid and significant transformations akin to microglia (37). In response to acute brain injury, OPCs migrate to the injury site, proliferate, and undergo morphological changes, such as hypertrophy, polarization or elongation (22, 38) (**Figures 1C, D**). The biological relevance of OPC morphological changes to their immune function is not yet clear. One possibility is that hypertrophy or polarization may enhance OPC proliferation and migration toward the lesion site, facilitating glial scar formation and blocking immune cell infiltration. Within 2–4 days post injury (dpi), a high density of OPCs occupies the center and surrounding area of the injury core (**Figure 1D**), and this density does not return to normal until 4 weeks after injury (22). These OPCs can be considered reactive OPCs, named after the reactive astrocyte. OPCs at the lesion site exhibit elongated morphologies and are polarized toward the lesion (22). These polarized cells appear to migrate to the lesion site within the first 4 dpi. In addition, the number of polarized OPCs peaks at 2dpi, coinciding with the peak of OPC recruitment to the lesion site. However, not all polarized OPCs migrate, indicating that polarization does not necessarily imply migration. Interestingly, a recent study using a combination of spatial and single-cell RNA transcriptomics identified a subset of OPCs (cluster 15) in the injury core and another group of OPCs (cluster 10) at the periphery (39). These cluster 15 OPCs may be the polarized cells. A combination of immunohistochemistry against OPCs, along with spatial and single-cell transcriptomics, could potentially address this hypothesis. Nevertheless, more studies are needed to further elucidate the link between OPC morphological changes and immune function.

Reactive OPCs migrate to the lesion site through the release of matrix metalloproteinase 9, an enzyme crucial for extracellular matrix degradation (40). Additionally, overexpression of Olig2, a key transcription factor for OPC fate determination, enhances OPC recruitment to the demyelinated lesions in the corpus callosum of lysolecithin-induced demyelination mouse model and promotes further differentiation (41) (**Table 1**). However, a subset of OPCs near the lesion site downregulates Olig2 expression after acute brain injury, but not in demyelinated area, and exhibit quiescent phenotype (6). These Olig2^low/neg^ OPCs may participate in glial scar formation or wound healing via distinct mechanisms (42).

Reactive OPCs play a crucial role in wound healing, as evidenced by the impairment of wound healing upon deletion of proliferating OPCs (22). This could be attributed to compromised communication between OPCs and microglia, as well as other infiltrating immune cells. The mechanisms underlying OPC-microglia communication following acute brain injury are not fully understood, but OPCs have been shown to modulate microglial homeostasis and reactivity during neuroinflammation (17). In a context of LPS-induced neuroinflammation, depletion of OPCs led to a significant increase in proinflammatory cytokines (IL-1β, IL-6, IL-12β, TNF-α, iNOS) in the brain. This did not involve periphery immune cells as the integrity of BBB remained unchanged after OPC depletion. This observation suggests that OPCs suppress microglial responses to LPS challenge. This immune-suppressive effect was mediated by transforming growth factor-β2 (TGFβ2) released by OPCs (**Table 1**), acting on TGFBR2 in microglia, which in turn regulates CX3CR1-mediated microglial immune responses (17). Interestingly, OPCs also change the expression of TGFβ2 after acute brain injury. Single-cell RNA sequencing of glial cells at the injury site has revealed that both clusters of OPCs (OPC1 and OPC2) upregulate *Tgfb2* mRNA (39). Hence, depleting reactive OPCs at the lesion site may enhance microglial response and inflammation, thereby impeding wound healing. Furthermore, OPCs interact with T cells in various pathological contexts (17, 24, 25, 27, 43) (further discussed in the next section). It is intriguing whether reactive OPCs repel peripheral immune cells after the initial response, a question that warrants further investigation.

The study from Koupourtidou et al. has revealed that a cluster of inflammatory genes, exceeding 140 in number, exhibited common alterations in all reactive glial cells surrounding the lesion, including microglia, OPCs, and astrocytes, following a stab wound injury in the mouse cerebral cortex—a model for traumatic brain injury (TBI) (39). Among the 241 upregulated genes, those involved in cell proliferation predominated. Intriguingly, a subset of each glial cell type, localized proximally to the injury site, exhibited a shared upregulation of genes associated with innate immunity pathways, such as type I interferon, CXCL10-CXCR3, and toll-like receptor (TLR2) mediated signaling pathways. This coordinated regulation of innate immunity pathways contributed to OPC accumulation at the injury site post-brain injury, without influencing OPC proliferation or altering the overall number of oligodendrocyte lineage cells near the injury site. However, whether this expression pattern is linked to immunoregulation or immunoprotection necessitates further investigation, as it did not induce new transcriptional states but rather modified inflammatory signatures through partial downregulation of inflammatory genes following stab wound injury.

## Multiple role-playing in multiple sclerosis

OPCs play a pivotal role in remyelination, particularly in demyelinating diseases like MS, prompting extensive research aimed at enhancing their proliferation and differentiation capabilities under inflammatory conditions. However, emerging evidence suggests that OPCs serve not only as precursors but also actively contribute to the generation and maintenance of the inflammatory milieu through bi-directional communication with immune cells. To date, studies investigating the interaction between OPCs and microglia in the context of MS are limited, with a greater focus on the communication between OPCs and peripheral immune cells.

### Recruitment and activation of peripheral immune cells by OPCs

Under normal physiological conditions, OPCs typically exhibit minimal expression of inflammatory genes. However, gene expression analysis of PDGFRα^+^ OPCs revealed increased expression of several cytokines and chemokines, eg. IL-1β and CCL2, in the lesions of MS patients as well as in the brains of cuprizone-induced demyelinated mice (25) (**Table 1**). IL-1β is known for its role in the induction, activation, and recruitment of CD4^+^ T-lymphocytes and T-helper cell type 1 (Th1) (30, 31), suggesting OPCs may recruit T cells to the lesion. Indeed, two independent transcriptomic studies have revealed immune-cell like OPCs in the spinal cord of experimental autoimmune encephalomyelitis (EAE) mouse model or at the chronic active lesion edge of MS patient brain (27, 44). A subset of OPCs in the EAE mouse model expressed genes involved in antigen processing and presentation via major histocompatibility complex class I and II (MHC-I and -II) (27) and the expression of MHC I and II could be induced by IFN-γ (24, 27) (**Table 1**). Interestingly, OPCs enhanced T cell survival in co-culture system, and this effect was further augmented when OPCs were pre-treated with IFN-γ. Furthermore, presence of OPCs increased the number of T cells expressing TNFα and IFN-γ, indicating that IFN-γ/MHC-II signaling between OPCs and T cells provide a positive regulatory feedback on inflammation. These studies strongly support the notion that OPCs are not only passive target of T cells but also actively modulate T cell function.

Interestingly, OPCs display similar alterations when exposed to an anti-inflammatory environment, such as IL4 and IL10. These changes include MHC-II expression and cytokine secretion (34) (**Table 1**). The expression of MHC I and II in OPCs induced by IFN-γ is likely mediated by STAT1 and Bach1 transcription factors. It has been shown that OPCs upregulate transcriptional factors such as STAT1 (signal transducer and activator of transcription 1) and Bach1 in response to the IFN-γ signaling, which in turn upregulates immune genes such as PSMB9 (proteasome 20S subunit beta 9) and TAP2 (antigen peptide transporter 2) in OPCs of mice and humans (45). PSMB9 aids in class I MHC peptide processing (46) and TAP2 facilitates antigen transport and MHC I folding (47).

OPCs in the demyelinated lesion increase the LRP1 expression, that enhances its ability to phagocytose and cross-present the debris (48). Knockout of LRP1 in OPCs notably reduced inflammation in demyelinating mouse models (EAE and cuprizone models). LRP1-deficient OPCs showed impaired phagocytic ability, lower levels of MHC-I, MHC-II, and immunoproteasome, ultimately resulting in enhanced myelin repair and neuroprotection (49). Hence, the conversion of OPCs to a pro-inflammatory phenotype may be mediated by LRP1.

OPCs also express antigens CD273 and CD274 (also known as programmed death ligand (PD-L) 2 and PD-L1, respectively) (24, 27, 43) (reviewed by Cabeza-Fernández et al (50),**).** When exposed to cerebrospinal fluid (CSF) from MS patients in the phase of progressive MS (pMS), OPCs upregulate PD-L1, which in turn suppresses T cell-induced inflammation (43). In addition, compared to CSF from patients in the relapsing phase, CSF from pMS patients reduces the MHC II and TNF-α expression, as well as the activation of NF-kB in OPCs, of MS, thereby suggesting OPCs exposed to pMS CSF impede T cell activation and proliferation (43). Hence, it’s tempting to speculate that OPCs adjust their phenotype in response to various conditions, thereby influencing T cell activity. This observation appears contradictory to the aforementioned findings, likely due to the comparatively simplified nature of *in vitro* systems compared to *in vivo* environment. The presence of myelin debris, CD4^+^ and CD8^+^ T cells, chemokines, and cytokines in MS lesions renders the signaling inputs to OPCs far more complex than those provided by CSF alone.

### Suicide cascade or heterogeneity of OPCs?

**Figure 2:**
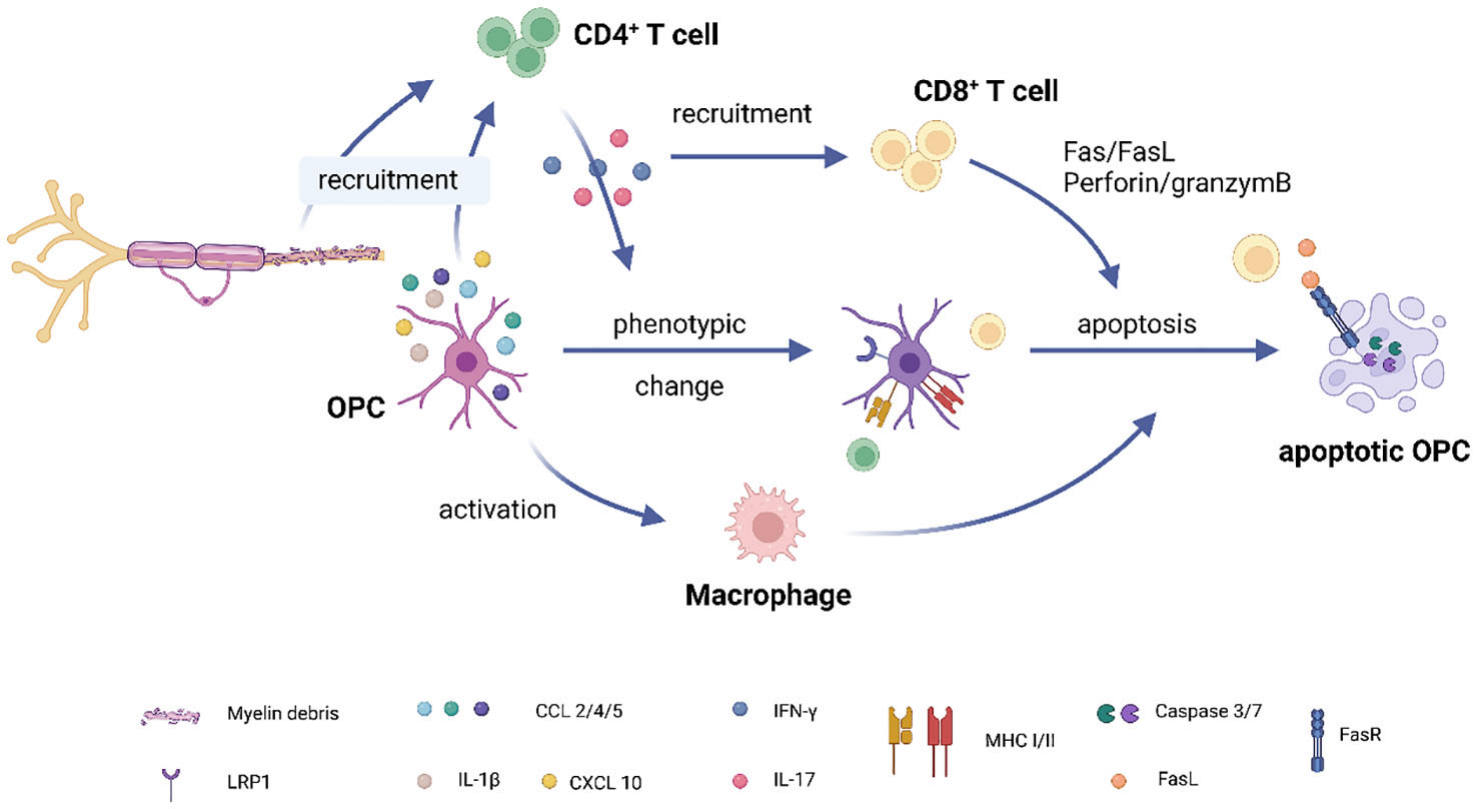
OPC-T cell interaction in multiple sclerosis. (1) Accumulation of myelin debris in demyelinated lesions attracts CD4^+^ T cells to the site. Additionally, OPCs express chemokines such as CCL2, CCL4, and pro-inflammatory cytokines like IL-1β, further aiding in the recruitment of CD4^+^ T cells. (2) Upon activation, CD4^+^ T cells migrate to the lesion site and release pro-inflammatory factors like IFNγ and IL17. (3) IFNγ plays a dual role: it recruits CD8^+^ T cells to the lesion while also inducing MHC expression in OPCs, thereby activating both CD4 and CD8^+^ T cells. (4) CD8^+^ T cells contribute to OPC apoptosis through pathways involving Fas/FasL and perforin/granzyme. (5) OPCs, in collaboration with CD4^+^ T cells, activate a subset of macrophages through mechanisms that are currently unknown. These activated macrophages subsequently induce OPC apoptosis. (Created with BioRender.com).

The number of OPCs is significantly reduced in MS lesions and normal-appearing white matter compared to control tissue (44, 51). This could be due to the peripheral immune cell induced OPC apoptosis. As mentioned above, OPCs activate T cells in the lesion and increase the number of IFN-γ producing T cells. This cytokine also attracts CD8^+^ T cells to MS lesions, resulting in OPC death through the activation of caspase cascades via the Fas/FasL and perforin/granzyme pathways (24) (**Figure 2**). Furthermore, a recent study observed that OPCs, along with Th1 cells, activate a subset of macrophages, leading them to become cytotoxic and subsequently induce OPC apoptosis (26). It seems that, initiated by unknown reason, OPC-expressed CCL4 starts to attract Th1 cells, and two together further recruit macrophages. In turn, the latter induce OPC and oligodendrocyte death, inducing demyelination (**Figure 2**). This self-destructive mechanism suggests that OPCs in MS may undergo a phenotypic change, becoming pro-inflammatory OPCs. However, it’s also plausible that the OPCs recruiting T cells and those undergoing apoptosis represent distinct subpopulations. OPCs exhibit heterogeneity based on factors such as their resident region, origin, age, and most importantly, their local microenvironment (5, 52–54). Kukanja et al. recently demonstrated that in the tissue of MS patients or mouse models, OPCs fall into two subtypes: homeostatic OPCs and disease-associated (DA)-OPCs (55). The latter subtype upregulates genes involved in immune function, such as B2m (coding Beta-2-Microglobulin), C4b (coding complement component 4b), and Igtp (Interferon gamma induced GTPase). Based on this evidence, we posit that DA-OPCs may primarily recruit immune cells in MS, while homeostatic OPCs mainly produce myelin and are the targets of immune cell-induced apoptosis. On the other hand, OPCs involved in remyelination can originate from either pre-existing OPCs through self-renewal or from neural stem cells (NSCs) (56, 57). The myelin sheaths formed by these two different-origin OPCs vary in thickness, with NSC-derived OPCs forming fully thick myelin sheaths and OPC-derived OPCs forming thinner sheaths (57). These results indicate that these OPCs exhibit distinct properties. Could it be that pre-existing OPCs are pro-inflammatory while NSC-derived OPCs are pro-myelinating? Further investigation is imperative to elucidate the processes leading to the accumulation of myelin debris and subsequent activation of immune responses, particularly the mechanisms through which myelin debris acts as an antigen, triggering immune cell reactions.

## Conclusion

OPCs have emerged as key players in various aspects of immune function, drawing significant attention in recent research. However, our understanding of how OPCs elicit immune responses remains limited. It is still an open question whether the immune-responsive OPCs represent a distinct subpopulation or are converted from homeostatic OPCs. There is an urgent need to identify the origin of immune-responsive OPCs, characterize them, and elucidate the cellular and molecular mechanisms underlying their contribution to CNS immune responses. The use of advanced techniques, such as *in vivo* two-photon imaging with transgenic animals and spatial- and single-cell RNA sequencing, will help address these questions and further enhance our understanding of the intricate interplay between OPCs and immune cells. Deeper exploration of these interactions holds promise for uncovering novel therapeutic avenues for multiple sclerosis and other neuroinflammatory disorders.

## Author contributions

AH: Writing – original draft. HS: Writing – original draft. LF: Investigation, Resources, Writing – original draft. JW: Writing – original draft. XB: Conceptualization, Funding acquisition, Supervision, Writing – original draft.
